# Deuterium trafficking, mitochondrial dysfunction, copper homeostasis, and neurodegenerative disease

**DOI:** 10.3389/fmolb.2025.1639327

**Published:** 2025-07-22

**Authors:** Stephanie Seneff, Anthony M. Kyriakopoulos

**Affiliations:** ^1^ Computer Science and Artificial Intelligence Laboratory, Massachusetts Institute of Technology, Cambridge, MA, United States; ^2^ Laboratory of Molecular Biology and Immunology, Department of Pharmacy, University of Patras, Rio-Patras, Greece; ^3^ Department of Research and Development, Nasco AD Biotechnology Laboratory, Piraeus, Greece

**Keywords:** deuterium, copper, histidine, amyloidogenic proteins, cardiolipin, mitochondrial dysfunction, neurodegeneration

## Abstract

Deuterium is a natural heavy isotope of hydrogen, containing an extra neutron. Eukaryotic organisms have devised complex metabolic policies that restrict the amount of deuterium reaching the mitochondria, because it damages the ATPase pumps, leading to release of excessive reactive oxygen species and inefficiencies in ATP production. Human metabolism relies heavily on the gut microbiome to assure an abundant supply of deuterium depleted (deupleted) nutrients to the host. Mitochondrial dysfunction is a hallmark of many chronic diseases, and deuterium overload, often due to gut dysbiosis, may be a major factor contributing to this issue. In this paper, we explore the potential role of certain amyloidogenic proteins, including amylin, amyloid beta, the prion protein, huntingtin, and *α*-synuclein, in disease processes that result in the accumulation of deposits of protein fibrils, along with lipid membrane components of damaged mitochondria, which we argue may be a mechanism to sequester deuterium in order to reduce the deuterium burden in the tissues. We show how cardiolipin, an anionic lipid synthesized in mitochondria and localized to the mitochondrial membrane, may play a central role both in trapping deuterium in the mitochondrial membrane and in inducing protein misfolding to facilitate the formation of deuterium-rich deposits. We focus on the potential role of the amino acid histidine and its interaction with the mineral copper, both to catalyze certain essential reactions and to facilitate the misfolding of amyloidogenic proteins triggered by contact with anionic phospholipids, particularly cardiolipin, and especially in the outer mitochondrial membrane of deuterium-damaged mitochondria.

## 1 Introduction

Deuterium is a natural nonradioactive isotope of hydrogen. It contains a neutron as well as a proton, making it twice as heavy, and this change alters its biochemical and biophysical properties, compared to hydrogen. Deuterium is present in seawater at 155 parts per million, which seems small, but, because hydrogen is so common, deuterium is actually present in the blood at 5 or 6 times the concentration of calcium, and its level is much higher than the level of trace minerals such as zinc and copper. Eukaryotic cells have developed complex metabolic strategies to severely restrict deuterium levels in the mitochondria, due to the fact that it damages the ATPase pumps, which produce adenosine triphosphate (ATP), the energy currency of the cell (Boros et al., 2024; Olgun, 2007; Seneff and Kyriakopoulos, 2025a; Seneff and Kyriakopoulos, 2025b).

Nicotinamide adenine dinucleotide (NADH) plays an essential role in supplying deuterium depleted (deupleted) protons to the mitochondrial intermembrane space, through the action of NADH dehydrogenase, a flavoprotein enzyme complex also known as Complex I. The proton that it delivers has been previously scrubbed of deuterium through the action of isomerases to exchange deuterium with hydrogen in the cytoplasmic water and techniques exploited by flavoproteins such as proton tunneling to support a high deuterium kinetic isotope effect (KIE) (Seneff and Kyriakopoulos, 2025a). The mitochondria are constantly producing deupleted metabolic water from oxygen via oxidative phosphorylation, capturing the deupleted protons that pass through the ATPase pumps from the intermembrane space into the matrix (Seneff and Kyriakopoulos, 2025a). Maintaining low deuterium levels in mitochondrial water is an essential aspect of metabolism, and a failure in this regard leads to severe mitochondrial dysfunction and oxidative stress, associated with many chronic diseases (Bhatti et al., 2017).

Cytochrome P450 enzymes (CYPs), primarily localized to the endoplasmic reticulum or the mitochondria, oxidize a variety of different substrates, replacing a proton with a hydroxyl group in the substrate, splitting molecular oxygen, and producing water as the other product. The first step is usually the abstraction of hydrogen from a C-H bond. Many studies have shown that CYPs typically have a high deuterium KIE for hydrogen abstraction, which means that the water molecule will be deupleted. The KIE can be greater than 10, suggesting the involvement of proton-coupled electron transfer and/or proton tunneling (Atkinson et al., 1994; Guengerich, 2017).

There is considerable recent interest in the idea of treating cancer with DDW (Lu and Chen, 2024). Long-term consumption of DDW can protect mice from cancer, and DDW has been shown to prolong survival of human cancer patients and to protect from relapse. Deuterium depletion improved the median survival time of glioblastoma multiforme patients (Somlyai et al., 2023). It has been hypothesized that a reduction in deuterium levels in the sugar-phosphates in the DNA backbone preserves the stability of hydrogen bond networks, protecting against aneuploidy and strand breaks (Boros et al., 2016). A study comparing 56 patients with pancreatic cancer who were treated with deuterium depleted water with 86 untreated control patients showed a strong, statistically significant Pearson correlation between survival time and length and frequency of DDW treatment (Boros et al., 2021).

Exposure of cells to deuterium-hydrogen ratios that differ from the natural ratio of 1/6600 alter the expression of hundreds of cancer-related and kinase genes, reflecting the fact that deuterium levels play a significant role in metabolic policy. One hundred percent of 124 cancer-related genes were upregulated in cultured cells exposed to 300 ppm deuterium (nearly twice the normal level) (Kovács et al., 2022). In fact, a case has been made that cancer cells paradoxically play a protective role in the organism by sequestering deuterium and releasing deupleted nutrients such as lactate to support mitochondrial recovery of resident immune cells (Seneff and Kyriakopoulos, 2025a). It has been hypothesized that eukaryotic cells must have a sensor for the D/H ratio in their environment that can act as a signaling mechanism to control metabolic policy (Yaglova et al., 2020).

Short chain fatty acids (SCFAs) are essential metabolites normally produced in abundance by the gut microbes, and they play a pivotal role in gut-brain communication and in maintaining human health (Silva et al., 2020). A recent study on the importance of metabolites produced by the gut microbiome in regulating neurological disorders defined the “gut-brain axis” as follows: “The microbiota gut-brain axis (MGBA) is a term used to describe the link between the gut and the brain involving the interplay of several systems, mainly the autonomic, central, and enteric nervous systems and the hypothalamic-pituitary axis” (Swer et al., 2023).

The interplay between hydrogen-producing fermentative bacteria and hydrogen consuming reductive acetogens, methanogenic archaea and sulfate-reducing bacteria becomes imbalanced in association with many gut disorders, such as inflammatory bowel disease (IBD), small intestinal bacterial overgrowth (SIBO), irritable bowel syndrome (IBS), and colon cancer (Carbonero et al., 2012). Interestingly, it has been shown experimentally that the molecular hydrogen gas H_2_, produced by hydrogenogenic microbes, is severely depleted in deuterium, relative to the levels of deuterium in the molecules from which it was extracted. It was found that microbially produced H_2_ contained only 20% as much deuterium as is normally found in water (Krichevsky et al., 1961). The acetogenic bacteria, such as *Faecalibacterium prausnitzii*, use H_2_ to reduce carbon dioxide to acetate, and to produce the derived SCFAs propionate and butyrate (Parsaei et al., 2021). These SCFAs, especially butyrate, are normally the primary nutrients for the colonocytes, and they can be expected to be extremely deupleted due to the deupleted protons they sourced from microbially-produced H_2_. An imbalance in the gut microbiome can lead to a deficiency in butyrate, which can cause metabolic stress for the colonocytes, and can even impact neurons in the brain via the gut-brain axis (Hodgkinson et al., 2023). Butyrate, functioning both as a histone deacetylase inhibitor and as a ligand for G-protein coupled receptors, can alter gene expression in the brain, preventing neurodegeneration and promoting regeneration (Bourassa et al., 2016).

In our recent publication, we developed a hypothetical but novel argument that there are a small number of organic molecules that may be uniquely configured to support deuterium trapping on carbon atoms (Seneff et al., 2025a). One class of molecules that we considered are those that contain an imidazole ring, most notably, the amino acid histidine. C_2_ in the imidazole ring is positioned between two nitrogen atoms, and it has a unique ability to exchange its proton with a deuteron in an irreversible spontaneous reaction. A study on peptide dendrimers containing histidine residues demonstrated experimentally that C_2_ of the imidazole ring of histidine was the only carbon atom in the dendrimer that was able to displace its proton with a deuteron when the dendrimer was immersed in heavy water. By contrast, all of the nitrogen and oxygen atoms were rapidly fully deuterated. However, once the molecule was placed in regular water under acidic conditions, all of the oxygen and nitrogen atoms immediately lost their bound deuterons, whereas the histidine residues retained theirs (Sheveleva et al., 2019). This property of the imidazole ring suggests to us the possibility that it might be useful in biological organisms as a deuterium trap. We have proposed that certain molecules that contain an imidazole ring, particularly one with a high pKa, may be able to permanently sequester deuterium, helping to reduce the deuterium burden in mitochondria (Seneff et al., 2025a).

Notably, the hydrogen/deuterium exchange (HDX) rate of a given imidazole ring depends on the concentration of OD^−^ (O(^2^H)^-^; the deuteroxyl anion that is the equivalent of a hydroxyl anion) in the medium, but it is also dramatically reduced when histidine residues are phosphorylated. The rate is also substantially slower in metal-bound histidine residues. Furthermore, the maximum rate of exchange occurs when the pH is high, and its value exponentially increases with the increase in the pKa of the imidazole ring itself (Miyagi and Nakazawa, 2024). Histidine residues that are buried within hydrophobic regions of the protein have poor access to water and therefore exchange very slowly. It is also conceivable that enzymatic action might greatly accelerate HDX rates. All these factors could play a role in controlling whether a histidine residue in a protein might be able to capture and secure a deuteron, given sufficient time, even under physiological conditions.

Another interesting class of carbon atoms that may serve as deuterium traps are the bis-allylic carbon atoms, notably present in both microbial breakdown products of haem and in polyunsaturated fatty acids (PUFAs). Bis-allylic carbon atoms are carbon atoms in a carbon chain that are single-bonded on both sides to carbon atoms that are double-bonded to their other neighbor. PUFAs, particularly those found in the mitochondrially-localized lipid, cardiolipin, are particularly attractive candidates for deuterium trapping. Cardiolipin is tightly integrated with the ATPase pumps in the inner membrane of the intermembrane space. It is plausible that it may be able to trap deuterons that pass through the membrane before they reach the pumps, serving as a shield or a filter (Seneff et al., 2025b).

In this paper, we develop some more new hypotheses concerning a potential role for PUFAs in protecting mitochondria from deuterium overload. In particular, cardiolipin, a novel phospholipid synthesized in the mitochondria, may serve to trap deuterium that enters the intermembrane space before it reaches the ATPase pumps. When cardiolipin becomes oxidized, it migrates to the outer membrane, where it can interact with cytoplasmic amyloidogenic proteins to facilitate the formation of pores in the mitochondrial membrane, and, ultimately, to trap mitochondrial debris from aged mitochondria within fibrillar extracellular deposits such as Lewy bodies (Seneff et al., 2025b). There is a compelling story around amylin, a protein whose misfolding is a core feature of diabetes. *α*-Synuclein, amyloid-*β*, and huntingtin are other proteins that may follow the same principles for trapping deuterium. We review the role of histidine residues, with particular emphasis on their interaction with copper, in facilitating the cascade of events that arise, we argue, due to severe deuterium overload in the mitochondria. Whether the imidazole rings in these amyloidogenic proteins can capture and sequester deuterium is an open question, but further experimental research will be necessary to resolve it. We will not address this question further in this paper, as peer reviewed papers on that topic are lacking.

## 2 Hydrogen peroxide in the peroxisome and the mitochondria: a source of deupleted water?

In this section, we will argue that hydrogen peroxide plays an important role in delivering deupleted water to the mitochondria, through a coordinated action between the peroxisome and the mitochondria. Furthermore, the supply of hydrogen peroxide from the peroxisome to the mitochondria increases as cells age (Titorenko and Terlecky, 2010).

Hydrogen peroxide (H_2_O_2_) is a non-radical reactive oxygen species that plays a powerful role as a signaling molecule to mediate redox reactions. The peroxisome is a hub for the H_2_O_2_ signaling network (Lismont et al., 2019). The peroxisome is responsible for the β-oxidation of long chain fatty acids and the synthesis of ether-phospholipids such as plasmalogens, bile acids and the PUFA docosahexaenoic acid (DHA) (Van Veldhoven, 2010). Many of the reactions that take place in the peroxisome ultimately reduce molecular oxygen to H_2_O_2_, and subsequently to H_2_O.

Catalase (CAT) is an essential enzyme in the peroxisome that converts two molecules of H_2_O_2_ into two molecules of water and one molecule of molecular oxygen. H_2_O_2_ is commonly produced as a consequence of the action of superoxide dismutase (SOD), which converts two molecules of superoxide and two protons from the water to H_2_O_2_ and molecular oxygen. Hence the net result of a cascade reaction of SOD and CAT is to convert 4 molecules of superoxide and four protons to 2 molecules of water and 3 molecules of molecular oxygen. This is an important process for protection from superoxide damage to tissues, but it also may serve to supply deupleted water to the organelles.

$$4O_{2}^{-}+4H^{+}\;\to \;2H_{2}O_{2}+2O_{2}\;\to \;2H_{2}O+3O_{2}$$

It can therefore be predicted that the water that is produced will be deupleted, for two reasons. First, when HDO ionizes, it is more inclined to split into OD^−^ and H^+^, rather than OH^−^ and D^+^, because deuterium binds more strongly to oxygen than does hydrogen. Therefore, protons in water will carry less deuterium than hydroxyls. More importantly, SOD has a very high solvent deuterium KIE, due to a proton-coupled electron transport mechanism. A study on a nickel-catalyzed SOD enzyme showed that it had a solvent KIE of 20 – favoring protons over deuterons by a factor of 20. The authors argued that a similar effect would be expected for CuZn-SOD, the SOD enzyme that operates in the peroxisome (Shearer, 2013).

Mitochondria and lysosomes interact through direct membrane contact sites, enabling bidirectional communication and regulation of both organelles. The contacts are dynamic, constantly forming and then breaking. Impairments in the formation of these contacts are associated with age-related diseases, specifically neurodegeneration and lysosomal storage diseases (Rizzollo and Agostinis, 2025). Membranes of organelles represent a diffusion barrier for H_2_O_2_, but do not block its transport (Laporte et al., 2020). Since H_2_O_2_ freely diffuses across membranes, it is easy for H_2_O_2_ produced in the peroxisome to reach a mitochondrion in close contact. In mitochondria, treatment with H_2_O_2_ rapidly, but in an indirect way, inactivates mitochondrial aconitase, an essential enzyme in the citric acid cycle (Bulteau et al., 2003). Evidence that the peroxisomal H_2_O_2_ reaches the mitochondria comes from experiments that showed that the suppression of CAT in the peroxisome decreased the mitochondrial aconitase activity by 85% within 24 h (Walton and Pizzitelli, 2012).

The mitochondria normally have a good defense mechanism against H_2_O_2_, which involves the oxidation of glutathione by glutathione peroxidase and its subsequent reduction by glutathione reductase. Glutathione reductase delivers one proton from the solution and the other from NADPH to the two sulfur atoms of glutathione disulfide (GSSG). Mitochondrial NADH kinase converts NADH to NADPH, consuming one ATP molecule in the process. Since mitochondrial NADH carries a deupleted proton (which is delivered to the intermembrane space by NADH dehydrogenase), the proton in NADPH will also be deupleted (Seneff and Kyriakopoulos, 2025a). Furthermore, glutathione reductase is a flavoprotein, and flavoproteins generally have a high deuterium KIE, as has been confirmed experimentally for glutathione reductase (Vanoni et al., 1990). What this all means is that the water molecules that are produced by glutathione reductase will be deupleted.

In general, oxidative stress mediated by H_2_O_2_ is considered as a key pathogenesis factor in neurodegenerative diseases. Relevant studies on the use of deuterium depleted water (DDW) against H_2_O_2_-mediated oxidative stress show that cells pretreated with DDW resist apoptosis, have a reduced production of ROS, and show increased activities of SOD, Cu/ZnSOD and CAT when exposed to ROS (Wu et al., 2020). This indicates that cells, and specifically mitochondria, that suffer from deuterium overload would be more susceptible to oxidative stress mediated by H_2_O_2_. It would therefore be essential for the cell to use the mechanism of deupletion proposed in this section to provide neuroprotection and counterweigh neurodegeneration mediated by deuterium overload.

Thus, we can argue that one important role that the peroxisome plays is to extract deupleted protons from solution and deliver them to the mitochondria, and, ultimately, to the ATPase pumps, in the form of H_2_O_2_. The mitochondria provide two more deupleted protons to synthesize metabolic water.

**FIGURE 1:**
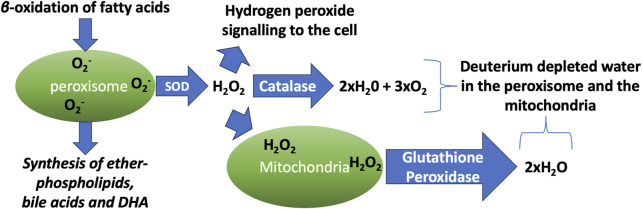
Schematic illustration of the processes that take place in the peroxisome and the mitochondria, which produce vital metabolites while generating abundant deuterium depleted water. DHA: docosahexaenoic acid. SOD: superoxide dismutase.

Figure 1 schematizes the process by which the peroxisome generates abundant deuterium depleted water through β-oxidation of fatty acids, followed by its conversion to deupleted water and oxygen via the catalytic activity of SOD and CAT in the peroxisome, as well as glutathione peroxidase in the mitochondria.

Age-associated mitochondrial dysfunction is regarded as a pivotal factor in the aging process. As cells age, CAT forms a tetrameric molecule in the cytosol and remains inactive. This results in increased levels of H_2_O_2_ in the peroxisome, which can then damage mitochondria, particularly if they have insufficient antioxidant defenses, such as depleted glutathione levels (Walton and Pizzitelli, 2012). However, this increase provides a mechanism to deliver deupleted water to the mitochondria, which may be a beneficial aspect to compensate for insufficient synthesis of metabolic water by the ATPase pumps, due to deuterium overload.

## 3 Mitochondria, cardiolipin, pyroptosis, and deuterium

Mitochondria serve broad functions in the cell, including cellular metabolism, calcium buffering, signaling pathways and regulating apoptotic cell death. Cardiolipin is a unique glycerophospholipid that is synthesized in the mitochondrial matrix and then inserted into the inner membrane of the intermembrane space. There, it stabilizes protein complexes and facilitates oxidative phosphorylation, and it also regulates the release of cytochrome c from mitochondria to launch an apoptotic cascade. Upon oxidation, cardiolipin migrates to the outer membrane, where it can come into close contact with amyloidogenic proteins in the cytoplasm. The interaction of cardiolipin with amyloid oligomers favors pore formation in the membrane, which causes osmotic swelling, loss of the voltage potential, and release of pro-apoptogenic factors. Besides the amyloidogenic proteins, several other proteins have also been shown to be able to punch holes in mitochondria through interaction with cardiolipin, including members of the Bcl-2 family, cobra venom cardiotoxins and bacterial toxins (Vassallo, 2025).

Pyroptosis is a cell death program that involves disruption of the plasma membrane and the release of pro-inflammatory cytokines into the external milieu. Gasdermin D (GSDMD) is a central protein controlling pyroptosis. It is activated by caspase-1, and it forms pores in the plasma membrane, inducing cell lysis (Liu et al., 2016). In response to endotoxemia, cardiolipin becomes oxidized by reactive oxygen species produced by complex II in cardiomyocytes. Oxidation and externalization of cardiolipin then induces GSDMD oligomerization and pore formation in mitochondria, a very early stage in the pyroptosis cascade (Tang et al., 2024).

Cardiolipin is tightly associated with ATPase pumps (Eble et al., 1990). It has been proposed that it buffers protons as they enter the membrane (Haines and Dencher, 2002). However, it may also be the case that it is able to trap and sequester deuterons before they reach the ATPase motor, where they can cause a stutter and induce reactive oxygen release. In neurons, cardiolipin is especially enriched in long-chain PUFAs, particularly docosahexaenoic acid (DHA), which has five bis-allylic carbon atoms. Bis-allylic carbon atoms are defined as carbon atoms in a chain that are single-bonded to the left and to the right to carbons that are double-bonded to their other neighbor: -C=C-C*-C=C-. These carbon atoms are unusual in that they are far more willing to give up their bound protons than carbon atoms in other configurations. They are the carbons in PUFAs that initiate and sustain the peroxidation chain reaction that produces a number of reactive products with a powerful role in the inflammatory response. In each step in the cascade reaction, a bis-allylic hydrogen is removed by a hydroxyl radical, and molecular oxygen is then added to form a highly reactive lipid peroxyl radical that propagates the chain reaction (Ng et al., 2022).

Remarkably, PUFAs that are deuterated at their bis-allylic carbon atoms have been found to be highly effective at quenching the chain reaction and suppressing the inflammatory response. Deuterium doping of PUFAs can have a profound effect in protecting from inflammation, as has been shown experimentally (Li et al., 2022). Cardiolipin may be able to situate itself in the inner membrane in such a way as to be able to eventually populate its bis-allylic carbon atoms with deuterium, and in this fashion protect the mitochondrion from oxidative damage (Seneff et al., 2025a). This also implies that, once it migrates to the outer membrane, it may have succeeded in permanently trapping one or more deuterium atoms, because deuterons bound to bis-allylic carbon atoms very rarely exchange back with hydrogen (Navratil et al., 2018).

Persistent accumulation of damaged mitochondria due to impaired mitophagy is a likely antecedent to neurodegenerative disease. Cardiolipin externalization activates mitophagy, and this is essential for rapidly sequestering injured mitochondria for clearance in the autophagosomes (Kagan et al., 2016). A team of researchers in Beijing, China conducted elegant experiments involving PrP106-126 exposure to mouse neuroblastoma cells grown in culture. They found that inhibition of the externalization of cardiolipin during treatment with Prp106-126 peptide aggravates mitochondrial oxidative stress by significantly intensifying the dysfunction of complex I and complex III. This demonstrates that externalization of cardiolipin affords protection from oxidative stress (Yang et al., 2023).

## 4 The two faces of lipid peroxidation: Good or bad for human health?

Peroxidation of lipids is a physiological process continuously ongoing in the organism. When it extends beyond limits, however, it leads to a wide spectrum of disease pathogenesis. The products of oxidized lipids, especially those derived from the PUFAs in lipid membranes that are attacked by reactive oxygen species (ROS) and reactive nitrogen species (RNS) are the ones that produce harm (Ramana et al., 2019). Unambiguously, the main diseases produced by excessive lipid peroxidation range from neurodegenerative diseases (manifesting as Alzheimer’s disease (AD), Parkinson’s disease (PD) and others), cardiovascular diseases (manifesting as atherosclerosis, ischemia and heart failure), autoimmune disorders, cancer, kidney and respiratory disease, and other inflammatory and immunological disorders (Ramana et al., 2019).

However, while excessive peroxidation of lipids is implicated in the pathogenesis of myriads of diseases, the same peroxidation of lipids provides the necessary signalling molecules for the regulation of very important cellular functions that incorporate the balance and regulation of autophagy (Gęgotek and Skrzydlewska, 2024) and the decision on survival or cell death (Zheng et al., 2024). Autophagy, i.e., the internal mechanism for recycling, biosynthesis and degradation of constituents and organelles via lysosomes, is probably the most essential universal process for the survival of all kinds of cells (He and Klionsky, 2009). Central to an organism’s homeostasis is regulated cell death. It is the same kinds of ROS and NOS modalities that oxidize and alter the structures of lipids in cellular membranes that signal for various kinds of cell death, apoptosis, ferroptosis, pyroptosis and many other death pathways (Zheng et al., 2024). Deviations in this important signalling disbalances the homeostasis that protects from tumorigenesis, infection and excessive forms of inflammation (Lee et al., 2023).

The study of A. Ayala et al. excellently describes the signalling molecules generated by lipoxygenase, cyclooxygenase and cytochrome-P40 peroxidation activities on lipids that regulate the whole of an organism’s homeostasis, from blood pressure to promotion of cell survival and proliferation, to reproduction and metabolism (Ayala et al., 2014). Under physiologic lipid peroxidation activities, the cells promote their antioxidant activities to remain alive and functional. It is only when the process of lipid peroxidation accelerates beyond the normal limits that the cells do not have the necessary repair mechanisms to survive the oxidative damage and therefore die (Volinsky and Kinnunen, 2013).

A good example of a PUFA lipoxygenase-peroxidation secondary end product that regulates the survival or the death of cells is 4-hydroxynonenal (4-HNE). 4-HNE in high concentrations is one of the most toxic and mutagenic metabolites of lipid peroxidation for the cell (Esterbauer et al., 1990). It is accused of causing mitochondrial dysfunction that mediates premature ageing, diabetes, and neurodegeneration, leading to AD (Bartman et al., 2024). However, at normal concentrations, 4-HNE is a major cell survivor-transcription factor regulator. It induces nuclear erythroid 2-related factor (Nrf-2), activating protein-1 (AP-1), the family of nuclear factor kappa-light-chain-enhancer of activated B cells (NF-κB), and the peroxisome proliferator-activated receptors (PPAR). Moreover, it activates the mitogen-activated protein kinases (MAPK) and Epidermal Growth Factor Receptor (EGFR)/Akt pathways, and protein kinase C. In this way the formation and release of 4-HNE through lipid peroxidation orchestrates the anti-oxidant-cell stress response, which is an ancient cellular defense mechanism, from microbes to humans, for survival and to counterweight disease (Nicolaou et al., 2010; Rath and Haller, 2011). Apart from cellular antioxidant defense, 4-HNE is a major regulator of normal proliferation and the cell cycle. Importantly, there are numerous studies showing, through the regulation of the aforementioned transcription factors and more, that 4-HNE causes cell cycle arrest and inhibits the proliferation of malignant cells through the p53 and p21 dependent pathways (for review see (Ayala et al., 2014)). Furthermore, in the same review, several studies describe the importance of normal 4-HNE concentrations in the regulation of autophagy and senescence.

Another important signalling metabolite that is generated through the cyclooxygenase-mediated peroxidation of PUFAs is malondialdehyde (MDA). MDA is a relatively stable oxidizing molecule that shows similar cytotoxicity and mutagenic potential with 4-HNE, reacting with various proteins and biomolecules. MDA is often used as an oxidative stress biomarker for the prognosis of various pathologic conditions (including cancer) (Gaweł et al., 2004; Fraile-Martínez et al., 2023). However, low levels of MDA are significant for the regulation of gene expression. These studies indicate that moderate levels of MDA are important for the regulation of insulin secretion (Wang et al., 2014), and the production of collagen (García-Ruiz et al., 2002). Significantly the transcription factors SP1 and SP3 that are influenced by MDA regulate the maturation of a cell and are involved in apoptosis and cell cycle arrest (chromatin remodelling). Their loss leads to severe and lethal forms of thrombocytopenia (Meinders et al., 2015).

Summarizing, although the peroxidation of lipids is responsible for the development of many diseases, it is a natural process and moderate to low levels of products have beneficial effects for the organism’s health, holding the wheel for a regulated autophagy, cell proliferation, haematologic homeostasis and a balanced programmed cell death. One way of reducing or even diminishing the capability of PUFAs to become oxidized is to deuterate them, i.e., to replace and exchange their hydrogen atoms with deuterium. *In vitro* and *in vivo* studies in animals have shown that the use of deuterated PUFAs can lower the peroxidation effects on producing atherosclerosis, control hyperlipidaemia, and reduce cholesterol levels in blood (García-Ruiz et al., 2002).

Although these are beneficial signals for human health, many questions are raised for the whole of the organism’s health since the deuterated lipids will accumulate in a vast number of cells and in their membranes, as in the case of myelin, and will cause a significant alteration in the metabolism of the deuterated host organism (Baumann et al., 2022). Most worrisome is the possibility that supplementation with pre-deuterated lipids will interfere with the ability of cardiolipin to filter out deuterons before they reach the ATPase pumps. This will have significant effects on essentials for life activities such as gestation. Moreover, the excessive accumulation of deuterated lipids in cell membranes will alter their physiologic characteristics of thickness and lamellar spacing (structure of membranes), and this is tightly linked to the development of neurodegeneration, infectious disease pathology, and protein lipid tumorgenicity amongst many others (Torres et al., 2021). These are similar pathological factors that are tightly regulated by the forementioned secondary peroxidation PUFA signalling metabolites 4-HNE and MDA, when their concentrations are moderate to low.

It would therefore be crucial to re-think the use of deuterated lipids to counterweight lipid, especially PUFA, peroxidation, as their application may seem promising but can hide severe and unpredictable pathologies raised in the recipient host. This is especially concerning for humans, as the molecular signalling in human disease is far more complex than in many animal models (Akhtar, 2015).

**FIGURE 2:**
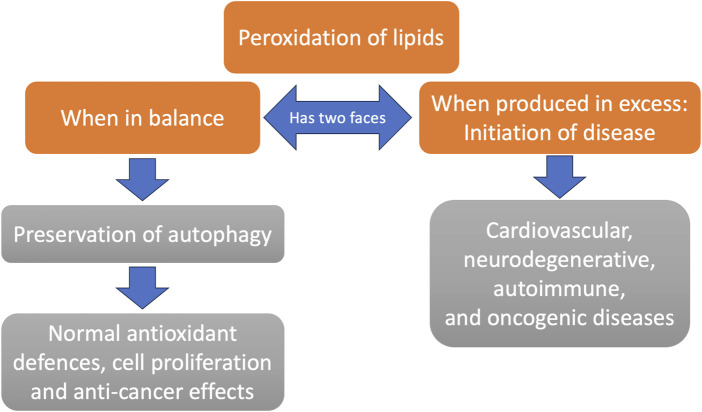
The two faces of lipid peroxidation. While membrane lipid peroxidation is generally viewed as a disease process, there are powerful signaling mechanisms in place that lead to a healthy outcome (e.g., preservation of autophagy) unless it occurs in excessive amounts. Excessive membrane peroxidation is associated with many chronic diseases.

Figure 2 schematizes the two faces of lipid peroxidation.

## 5 Copper’s roles in the Mitochondria and in protein misfolding

Copper is a highly reactive metal, but it is an essential cofactor for several vital enzymes, notably two that are active in the mitochondria. Copper, zinc superoxide dismutase (CuZn-SOD) detoxifies superoxide by converting it to the less reactive hydrogen peroxide and oxygen. It depends on both copper and zinc as cofactors, but only copper regulates its activity (Harris, 1992). The copper ion is bound to either three or four histidine side chains in the enzyme (Hart et al., 1999). When exposed to high levels of its own reaction product, H_2_O_2_, CuZn-SOD’s activity is suppressed, via oxidation of His-118 to form 2-oxo-histidine (Uchida and Kawakishi, 1994).

Cytochrome c oxidase, also known as complex IV, at a final step in the respiratory chain, catalyzes the reduction of oxygen to water. In cytochrome c oxidase, the catalytic core is formed by three subunits containing three copper atoms. Two copper atoms are held in place in a bimetallic center in subunit 2 by six histidine residues that are highly conserved among multiple family members. A third copper atom is associated with the haem group of subunit 1 (Horn and Barrientos, 2008). Histidine residue 503 (His-503) is crucial for the enzyme’s catalytic action. A water molecule is securely fixed between His-503 and Asp-91 and is linked to two water arrays that reach the surface of the molecule. These two residues together trap the proton that is transferred from the surface through the water arrays. Once oxidized, His-503 releases the proton to Asp-91, and Asp-91 then donates the proton to the dioxygen reduction site (Muramoto et al., 2007).

Biological organisms have developed sophisticated strategies for copper transport and copper delivery to the enzymes that require it, but these mechanisms can become defective, leading to both copper toxicity and copper deficiency, sometimes even co-occurring. Min et al. wrote in the abstract of a paper published in 2024: “The pathophysiology of ALS [Amyotrophic Lateral Sclerosis] involves many signs of a disruption in copper homeostasis, with both excess free levels and functional deficiency likely occurring simultaneously” (Min et al., 2024). Copper interacts with the key aggregation-prone proteins associated with ALS, including TAR DNA binding protein 43 (TDP-43), CuZn-SOD, and fused in sarcoma (FUS). Mutant SOD1 aggregates more readily when copper is present (Tokuda and Y Furukawa, 2016). Unbound copper and excessive ROS can accelerate protein aggregation. This can lead to copper sequestration in misfolded fibrils along with copper deficiency in the mitochondria, causing impaired energy production (Min et al., 2024).

At least one histidine residue is present in the majority of amyloidogenic proteins, and these histidine residues interact with copper in important ways, influencing their conformational shape. Histidine is a unique amino acid with respect to its interactions with copper. A study on cultured HaCaT keratinocytes exposed to CuSO_4_ assessed the potential for each of the 20 coding amino acids to ameliorate copper toxicity (Ha et al., 2023). Although cysteine binds more strongly to copper than does histidine, histidine was the only amino acid that protected the cells from death due to copper overload. Despite its potent ability to chelate copper, cysteine had no cytoprotective effects. Cysteine is one of the three amino acids in glutathione, and it has been shown that glutathione greatly facilitates copper uptake by cells via its ability to bind copper (Maryon et al., 2013). But, unlike histidine, it is not able to protect from copper toxicity.

Amyloid beta (Abeta) is the peptide that is associated with Alzheimer’s disease. High levels of copper are accumulated near Abeta peptide-containing plaque in the brain. Several histidine residues are involved in Cu^2+^ coordination in Abeta. The N-terminus and His-13 are crucial for Cu^2+^ binding, and His-6 and His-14 are also implicated. Copper accumulates in the senile plaque where it is tightly bound to Abeta (Syme et al., 2004). This suggests that the plaque may serve a role in sequestering free copper to protect from its toxic effects. *In vitro*, the Abeta-Cu^2+^ complex, in the presence of a reducing agent, reduces oxygen to produce H_2_O_2_, and this involves recycling copper from Cu^2+^ to Cu^1+^ and back to Cu^2+^ along with the oxidation of the reducing agent (Jiang et al., 2010). Abeta has a very high affinity for Cu^2+^. It has been well established that Abeta, when incubated with copper and oxygen, produces H_2_O_2_, while reducing Cu^2+^ to Cu^1+^, during the period when it slowly transforms into soluble oligomers (Huang et al., 1999). However, in later stages of the incubation period, the H_2_O_2_, levels decrease, suggesting that the fibrils that are ultimately formed may clear the H_2_O_2_. Abeta also binds to zinc, but at a lower affinity, and it does not bind at all to manganese or iron (Mayes et al., 2014).

In 2014, Mayes et al. decided to specifically investigate the ability of Abeta(1–42) peptide fibrils to remove H_2_O_2_ and to determine the exact reaction mechanisms (Mayes et al., 2014). Incubation of Abeta(1–42) fibrils with Cu^2+^ at a one-to-one ratio with H_2_O_2_ revealed that these fibrils were able to clear the H_2_O_2_, while causing the formation of carbonyl groups in the peptide. Carbonyl formation is routinely exploited to detect the production of reactive oxygen species such as the hydroxyl radical. Thus, these authors concluded that Abeta fibrils may not be completely innocuous, as is widely believed (Mayes et al., 2014).

In early work, excess copper has been linked to neurotoxicity in association with PD (Cruces-Sande et al., 2019) and Alzheimer’s disease (X Li et al., 2023), and in a Parkinsonian model in studies on *C. elegans* (Weishaupt et al., 2024). A comprehensive review of copper’s role in neurodegenerative disease, published in 2024, found substantial evidence that disrupted copper homeostasis can be linked to AD, PD, Huntington’s disease, ALS, Wilson’s disease, Menkes disease, prion diseases, and multiple sclerosis (Zhong et al., 2024). There is a complex network of copper chelators and transporters that regulate cellular copper uptake, distribution, and metabolism (Zhong et al., 2024).

**FIGURE 3:**
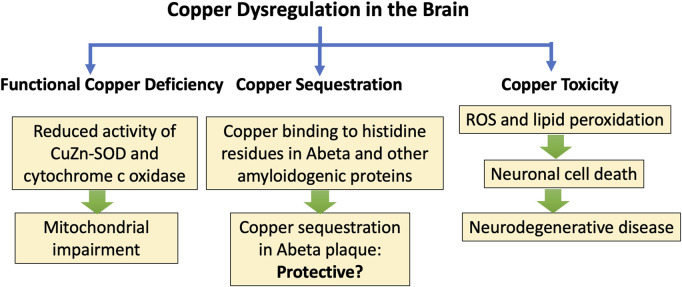
Schematic of processes leading to neurodegenerative disease and protein misfolding in association with impairments in copper transport and delivery. A proposed hypothesis is that copper sequestration in plaque regions may be protective.

Free copper is extremely reactive through Fenton chemistry, and it stimulates the secretion of pro-inflammatory cytokines such as interleukin-1 (IL-1), IL-4, and tumor necrosis factor α (TNFα) in both the brain and the blood (Tokuda et al., 2009). Figure 3 is a schematic of the processes by which impaired copper homeostasis can lead to neurodegenerative disease, and the hypothesis that copper trapping in plaque regions may be protective.

## 6 The role of copper-histidine binding in amyloidogenic prion proteins

Histidine residues of proteins are unique in their function. Due to their imidazole ring, they possess unique acid-base abilities and under pH 6 are usually in a state of protonation, i.e., being positively charged (Dokainish and Kitao, 2016). However, the acid-base dissociation constant (*Ka*) of imidazole rings of histidine residues often reflects the overall protein catalysis functions. The pKa, of imidazole rings varies according to the microenvironment that protein folds in, and this huge pKa fluctuation enables the protein’s histidine binding with metals (Miyagi and Nakazawa, 2024). One of the most biologically significant protein-histidine residue/imidazole binding is with copper (Cu^2+^). In fact, the Cu^2+^-histidine interaction (binding) in nature can match the importance of iron (Fe^2+^) binding with proteins, and the consequent haem interactions that support the very existence of life (Walton et al., 2023).

Intriguingly, the interactions of N-terminal histidine residues of the prion protein (PrP) mainly with Cu^2+^ sequestration and accumulation in diseased brain (T Frączyk, 2021) has long been identified to be responsible for the neurotoxic effects of PrP in neurons producing prion disease. Each of the eight amino acid sequences that are repeated four times in the octarepeat domain of the N terminal of PrP contains one histidine. The four histidine residues, through their imidazole rings, coordinate the occupancy and binding of one atom of Cu^2+^ at neutral pH (7.4) (Chattopadhyay et al., 2005). However, by all means, the binding of Cu^2+^ is not necessarily pathogenic since it has also been found to produce neuroprotection due to the *cis* interaction with the C terminal of PrP.

The histidine residues provide a tertiary structural interaction with the two domains of PrP and copper, that is also protective in function due to involvement of the conserved central region of the protein (Roseman et al., 2020). Moreover, recent experimental results show that two more histidine residues located in the C terminal region of PrP provide protection against neurotoxicity, when this is exerted from the N terminal region (Schilling et al., 2020). Also, histidine 96 and histidine 111 (human PrP numbering) that are located outside the octarepeat region, are also actively involved in PrP-Cu^2+^ binding (Sánchez-López et al., 2018). Therefore, Cu^2+^ coordination by the histidine residues in PrP is central to either neuroprotection or neurodegeneration. Conformational changes that correspond to the binding of PrP with Cu^2+^ are associated with prion disease, and these are usually induced by alterations to Cu^2+^-to-histidine occupancy within the PrP molecule. These structural deformations occur during native PrP (PrPc) misfolding to give the pathogenic PrPsc variant that produces amyloidogenesis (Sánchez-López et al., 2018).

Native human PrP can bind up to six Cu^2+^ ions, in a manner involving all the imidazole groups of the histidine residues located in the N-terminal repeats as well as His96 and His111. The prerequisite for this interaction is that, in these locations of the PrPc backbone, two of the amide groups are deprotonated. This makes the histidine-Cu^2+^ interactions and subsequent conformational changes of PrPc upon Cu^2+^ binding pH dependent (Staś et al., 2023). Moreover, the histidine imidazole Cu^2+^ binding (anchoring) is followed when Cu^2+^ is in a coordinated state, and this is achieved by the presence of two deprotonated backbone amides (Rivillas-Acevedo et al., 2013). The same counts when the occupancy of Cu^2+^ involves less than 6 ions. In this situation, multiple histidine residues of the octarepeats are involved in the binding reaction. However, the involvement of a deprotonated amide chelation reaction preceding the Cu^2+^ anchoring is still necessary. Moreover, other histidine residues of the neighboring PrPs can be involved in Cu^2+^ anchoring, with an end result of PrP formation of oligomers (Yang et al., 2008). When the α-helices of PrPc refold into β-sheets, this gives the pathogenic PrPsc misfolded variants, and then the prion proteins become pathogenic, resulting in neurodegeneration (Derreumaux, 2001).

## 7 *Akkermansia*, butyrate, GLP-1, diabetes and obesity

In this section, we will describe the role that the gut microbes, particularly *Akkermansia muciniphila*, play in protecting the host from diabetes and obesity, via the release of a protein that induces the secretion of glucagon-like peptide 1 (GLP-1) from intestinal L-cells (Rodrigues et al., 2022). This is important not only because GLP-1 receptor agonists have become very popular as pharmaceutical drugs to treat diabetes and obesity (JY Wang et al.), but also because, as we will see later, GLP-1 prevents amyloidosis of the amyloidogenic small peptide hormone amylin, which is normally released along with insulin from the pancreatic β-cells when blood sugar levels are elevated. Amylin misfolding is a strong feature of type II diabetes (Asthana et al., 2018).

*A muciniphila* is a commensal bacterium in the human gut that feeds only on the sulfomucins produced by the colonic goblet cells. It breaks down the sulfomucins into small organic metabolites that can then be used by hydrogenogenic bacteria, mainly *Bacteroidetes* and *Firmicutes* strains, as a source for hydrogen gas production through fermentation (Wolf et al., 2016). The hydrogen gas that is produced is then used as a reducing agent to convert carbon dioxide into organic matter, primarily the three major SCFAs, acetate, propionate and butyrate. This process of hydrogen gas recycling is a crucial component of gut microbial metabolism that is essential for the wellbeing of the host. *A. muciniphila* colonizes the infant gut shortly after birth, and, in the healthy gut, their colonization is maintained throughout the lifespan. Butyrate is a favored fuel of the colonocytes (Salvi and Cowles, 2021), likely in part because it is expected to be an excellent source of low-deuterium protons. Butyrate is also passed into the circulation from the gut, so it or its derivatives can also fuel mitochondria in distant tissues, including the brain (Bourassa et al., 2016).

There is an inverse correlation between the abundance of *A. muciniphila* in the gut and metabolic disease, and there is recent excitement among practitioners about the potential of *A. muciniphila* probiotics to treat several different metabolic diseases, including obesity, type II diabetes, cardiovascular disease, and nonalcoholic fatty liver disease (Yan et al., 2021; Rodrigues et al., 2022). Despite the fact that it consumes sulfomucins, its presence in the gut is usually associated with a greater abundance of sulfomucins, perhaps because it facilitates the supply of butyrate to the colonocytes, supplying them with the energy they need to produce sulfomucins, and also, importantly, maintaining low deuterium levels in the mitochondria of the colonocytes (Seneff and Kyriakopoulos, 2025b).

*A. muciniphila* secretes a protein called P9 that has been shown to upregulate the expression of glucagon-like peptide-1 (GLP-1), an incretin hormone secreted by the intestinal L-cells (Buteau, 2008). GLP-1 has many positive effects on gut homeostasis, including stimulation of insulin secretion, slowing of gastric emptying, appetite suppression, and increasing β-cell proliferation (Buteau, 2008; Müller et al., 2019). GLP-1 receptor agonists are a promising therapy for diabetes and have also recently enjoyed extraordinary success as a therapy to support weight loss, by reducing the appetite (Wang et al., 2023).

GLP-1 is known as an insulinotropic hormone, because it induces the release of insulin from pancreatic β-cells in response to rising serum glucose levels (Müller et al., 2019). In a study on a rat insulinoma cell line, a commonly used model for pancreatic *β*-cells, treatment with GLP-1 induced mitochondrial biogenesis, through a mechanism that involved increased cAMP activation. The expression of a key regulator of mitochondrial biogenesis was increased dramatically after 1 h of treatment of the cultured cells with GLP-1, and the expression level remained elevated for 48 h after treatment (Kang et al., 2015). Methylglyoxal is a highly reactive glycating agent produced as a by-product of glycolysis, and excess exposure can induce apoptosis in pancreatic *β*-cells. GLP-1 was shown in an *in vitro* experiment to protect *β-*cells from methylglyoxal-induced apoptosis, by improving mitochondrial function and suppressing prolonged activation of AMP-dependent protein kinase (AMPK) (Chang et al., 2016).

Upon glucose stimulation, the pancreatic *β*-cells release insulin into the circulation from secretory granules, where it is temporarily stored, along with another short peptide called amylin (islet amyloid polypeptide; IAPP), a 37-residue peptide hormone that slows gastric emptying and promotes satiety. In type II diabetes, the number of docked granules is about 1/3 lower than in normal cells, and the rate of glucose-stimulated exocytosis is only 20% of that in normal cells (Gandasi et al., 2018). Remarkably, GLP-1 mobilizes insulin secretory granules to dock at the plasma membrane, which then increases the rate of release of insulin from the granules (Kwan and Gaisano, 2005). IAPP is of great interest here, because, like amyloid β, it is an amyloidogenic protein (Press et al., 2019). In the next section, we review what is currently known about IAPP and its critical role in pancreatic β-cell destruction and type II diabetes.

## 8 IAPP and diabetes

Type II diabetes can be characterized as a protein-misfolding disease, and the protein involved in its pathology is hIAPP/amylin. More than 90% of patients with type II diabetes have amyloid deposits of IAPP in their pancreas, as determined by post-mortem studies. As discussed previously, monomeric hIAPP binds to specific receptors in the membranes of cells to regulate glucose homeostasis and gastric emptying (Engel, 2009).

hIAPP has several positively charged residues on its N-terminal side, which are involved in the interaction of hIAPP with negatively charged lipids (Engel, 2009). His-18 is central to the misfolding potential of hIAPP. In fact, the isoform found in both rats and mice is missing His-18, and their versions of the protein are immune to the formation of toxic oligomers or fibrils (Asthana et al., 2018). Mice with transgenic overexpression of human IAPP develop toxic IAPP oligomers intracellularly within the secretory pathway, whereas overexpression in mice of rat IAPP does not (Gurlo et al., 2010). His-18 is important for both fibril formation and membrane interaction. Protonation of His-18 is crucial for its interaction with and entry into lipid membranes, and this leads to membrane disruption and toxicity (Cao et al., 2013).

Cu^2+^ binds to amylin, promoting the formation of toxic oligomers. Cu^2+^ induces a conformational change into a more compact structure containing α-helices that resists HDX and is protected from enzymatic hydrolysis by insulin degrading enzyme (IDE). Preincubation with copper sulfate increases its toxic effects on neurons (Sinopoli et al., 2014). An imidazole nitrogen atom in His-18 is the main binding site for Cu^2+^ (Magrì et al., 2016). Membranes, especially those with an abundance of negatively charged lipids, are able to catalyze hiAPP transformation into an α-helical structure, especially in the presence of free copper, which then leads to membrane penetration and pore formation (Engel, 2009). Anionic lipids drastically accelerate aggregation, an effect that is substantially weaker for sphingophospholipid and zwitterionic phospholipids (Sitton et al., 2025). Even low levels of anionic lipids promote membrane permeabilization due to pore formation. Cholesterol at physiological levels significantly reduces the rate of amyloid formation and protects membranes from leakage (Zhang et al., 2017).

A study on human kidney embryonic cells grown *in vitro* revealed that culturing the cells with copper and amylin resulted in greater stress to the cells than culturing with amylin alone. Multilayer lamellar compact structures were observed on the cell membranes and were associated with amylin aggregates. Copper ions were localized only in the vicinity of the amylin aggregates. Fibrillar structures were also observed, but they were not associated with copper ions, suggesting that copper promotes the formation of toxic oligomers that disrupt membranes (Congiu et al., 2021).

Impaired autophagy plays an important role in IAPP toxicity, due to the accumulation of excessive amounts of IAPP oligomers intracellularly. Autophagosomes can efficiently dispose of IAPP small oligomers in p62-containing vacuoles to keep them from becoming toxic (Gupta and Leahy, 2014).

A seminal paper by Gurlo et al. published in 2010 provides tremendous insight into the early steps in the misfolding of IAPP that eventually leads to β-cell destruction. These authors found that toxic IAPP oligomers initially appear within the granules in the secretory pathway, where they disrupt the membranes of the granules, leading to their release into the cytoplasm. They then come into close proximity with mitochondrial membranes, which they also penetrate and disrupt. The destruction of the mitochondria may indeed be an early step in the pathology, eventually leading to β-cell death and a reduction in the number of functioning β-cells. These authors used antibody labelling of islets isolated from hIAPP transgenic mice to confirm the hypothesis that toxic IAPP oligomers form at all steps of the secretory pathway. They also confirmed that toxic oligomers were identified intracellularly in β-cells from humans with type II diabetes. Mitochondria adjacent to the oligomers exhibited disrupted membranes, whereas mitochondria that were remote from the aggregates were relatively normal. The authors concluded in the abstract that “IAPP toxic oligomers are formed intracellularly within the secretory pathway in T2DM. Most striking, IAPP toxic oligomers appear to disrupt membranes of the secretory pathway, and then when adjacent to mitochondria, disrupt mitochondrial membranes” (Gurlo et al., 2010).

**FIGURE 4:**
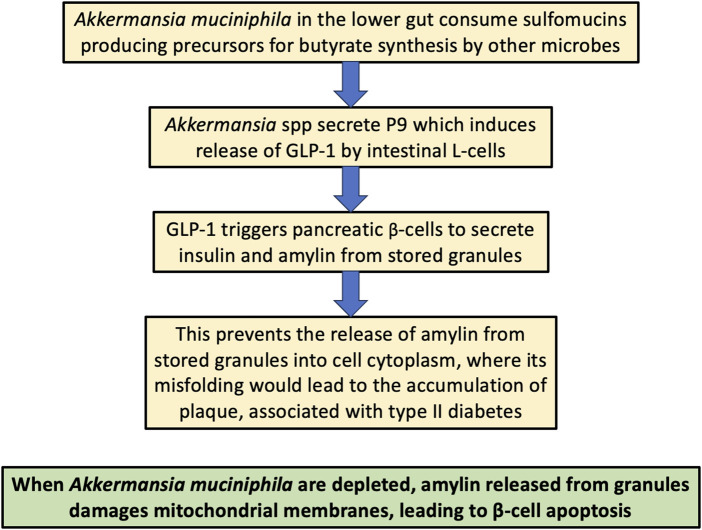
Schematic of the process by which *Akkermansia muciniphila* in the gut protect the host from type II diabetes, both through supplying deupleted butyrate to the colonocytes and by stimulating L-cells to release the hormone GLP-1, which in turn facilitates the release of amylin from stored granules into the extracellular space, protecting from its build-up in the cytoplasm, which can lead to destruction of mitochondria and β-cells through its amyloidosis.

Figure 4 illustrates schematically the process by which *Akkermansia spp* protect the host from type II diabetes. This is one of the best examples available of a direct pathway between the gut microbiome and disease caused by protein misfolding.

Mitochondria are central to the process that couples glucose metabolism to insulin exocytosis, and their dysfunction is an early feature of diabetes (Supale et al., 2012). Mitochondrial autophagy (mitophagy) is critical for the clearance of damaged mitochondria, and it is primarily regulated by two proteins, PTEN-induced putative kinase 1 (PINK1) and the E3 ubiquitin ligase Parkin. Genetic mutations in these proteins are linked to PD, but they have also been found to play a role in diabetes pathogenesis and associated β-cell dysfunction (Qu et al., 2011; Jin and Youle, 2012).

The antioxidant defenses of β-cells are weak compared to other cell types, which leaves them more susceptible to oxidative stress (Kaufman et al., 2015). 12-Lipoxygenase overexpression in pancreatic islets induces oxidative stress, which can damage membrane lipids through a lipid peroxidation chain reaction (Tersey et al., 2014). We will have more to say later in this paper about mitochondrial membranes and how they are altered in response to oxidative stress, specifically focusing on a potential role for the anionic lipid cardiolipin both in protecting the ATPase pumps from deuterium toxicity and in facilitating the removal of dysfunctional mitochondria through the action of amyloidogenic proteins.

## 9 Amyloid-beta and Alzheimer’s disease

Amyloid beta (Abeta) is the amyloidogenic peptide that is associated with Alzheimer’s disease. It is derived by cleavage of the transmembrane amyloid precursor protein (APP), and it is found as both a 40-peptide and a 42-peptide sequence. Abeta-42 is considered to be much more toxic than Abeta-40 (O'Brien and Wong, 2011). Amyloid plaque is primarily formed of Abeta fibrils, and much research on potential therapies has centered on creating anti-amyloid monoclonal antibodies, with the aim of clearing the plaque by targeting the peptide for degradation (Cummings et al., 2024).

A fascinating paper by Serra-Vidal et al. examined the dynamics of Abeta aggregation over time using HDX. They found that, at the beginning of the aggregation process, nearly all of Abeta-40’s amides had access to free exchange, whereas only about 33 of the 41 amides available in Abeta-42 underwent exchange. Over time, both forms experienced further reduction in the availability of amides for exchange, with Abeta-42 showing a faster timeline for reduction. They stated that this observation provided evidence that Abeta-42 is more prone to aggregation than Abeta-40. As time elapsed, they identified three phases where different populations became dominant. They associated the early phase with monomers, the middle phase with soluble oligomers, and the final phase with insoluble fibrils. In parallel, they tracked the neurotoxicity of the peptides, and neuronal viability showed a clear dip during the period when the oligomers were most prominent. This experiment was an elegant approach to demonstrating that it is likely the oligomers that are the toxic species (Serra-Vidal et al., 2014).

As early as 1993, it was proposed that Abeta has the ability to form channels within lipid membranes, and this may be its primary toxic effect (Arispe et al., 1993). Lipids are able to uniquely modify the secondary structure and toxicity of Abeta oligomers and fibrils. Zhaliazka et al. (2023) examined the effects of phosphatidylcholine, cardiolipin, and cholesterol on Abeta structure. They found that all three of these molecules strongly accelerated the rate of fibril formation compared to a lipid-free environment. They wrote: “Remarkably, under the optimized micelle conditions, Aβ42 assembles into oligomers that insert into lipid bilayers as well-defined pores and adopt a specific structure with characteristics of a β-barrel arrangement” (Zhaliazka et al., 2023). Both cardiolipin and cholesterol drastically increased *β*-sheet formation in Abeta-42 oligomers, which greatly increased the toxicity compared to lipid-free environments (Zhaliazka et al., 2023). Anionic cardiolipin had the strongest effect. By handling Abeta oligomers as if they were membrane proteins, Serra-Batiste et al. were able to demonstrate that Abeta-42 assembles into stable oligomers that get incorporated into membranes as pores, causing neurotoxicity (Serra-Batiste et al., 2016).

A remarkable study published in 2015 used extensive and sophisticated imaging techniques to assess whether there is a localized relationship between regions of the brain showing hypometabolism and regions with plaque buildup. The last sentence in their abstract read “Thus we conclude that regional fibrillar amyloid deposition has little to no association with regional hypometabolism.” (Altmann et al., 2015). Increasingly, researchers in the field are concluding that the fibrillar plaque is not toxic, and, in fact, may serve a useful role by clearing the toxic form of Abeta. This also explains why drugs that have been shown to reduce plaque burden do not actually improve disease symptoms, and they come with a significant side-effect burden (Karran and Hardy, 2014; Waite, 2015).

The transition metals copper, zinc and iron are all enriched in Abeta plaques. Abeta interactions with copper and zinc can control the aggregation state of Abeta. Abeta-Cu^2+^ complexes are redox-active, and therefore they can release reactive oxygen (Smith et al., 2006). The coordination sphere around a bound Cu^2+^ ion in Abeta resembles the active site of superoxide dismutase. Methylation of either of the nitrogen atoms in the imidazole rings of the histidine residues 6, 13 and 14 prevents formation of bridging histidine moieties. Remarkably, the modified peptides were four times more effective at generating H_2_O_2_ (behaving like a SOD), but they were not neurotoxic. This was probably due to the fact that the modification inhibited the interaction between the peptides and cell surface membranes. This suggests that the toxicity of Abeta may be due to its effects at the membrane (Tickler et al., 2005).

Abeta plaque accumulates in the extracellular space, but Alzheimer’s is also strongly associated with another misfolded protein, phosphorylated tau. Neurofibrillary tangles, containing abundant tau, accumulate *inside* the neurons. Tau pathology depends upon the presence of cardiolipin in the outer mitochondrial membrane. Tau oligomers bind to cardiolipin, and this binding leads to the creation of membrane pores, causing mitochondrial swelling, cytochrome c release, and a reduction in membrane potential (Camilleri et al., 2020). We therefore propose that it is plausible that the migration of oxidized cardiolipin to the outer membrane, due to excessive release of reactive oxygen species by the ATPase pumps, is the initiating factor in tau tangle formation and an early event in AD progression.

**FIGURE 5:**
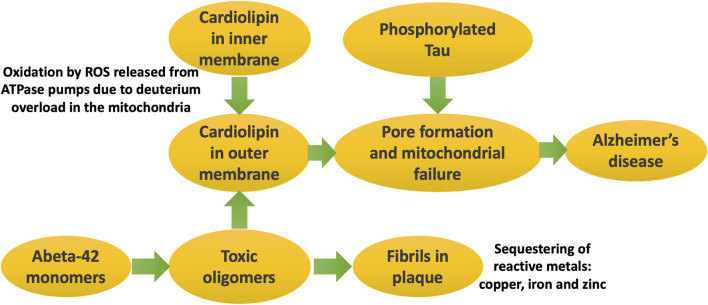
Schematic of processes that eventually lead to Alzheimer’s disease. It is the soluble oligomers that are the toxic form of Abeta. They disrupt the mitochondrial outer membrane, especially when cardiolipin has migrated there due to its oxidation by ROS generated during ATP synthesis. Phosphorylated tau forms pores in the mitochondrial membrane, triggered by cardiolipin accumulation there. Mitochondrial dysfunction in critical brain regions leads to symptoms of Alzheimer’s disease.

Figure 5 illustrates schematically the processes that eventually lead to Alzheimer’s disease, where the major players are Abeta, phosphorylated tau, and cardiolipin.

## 10 Huntingtin and Huntington’s disease

Mitochondrial dysfunction plays a major role in neurodegenerative diseases, including Huntington’s disease, AD, and PD. Disruption of synaptic connectivity and impaired neuronal signaling are early signs of these diseases, and this could be due to defective processing by synaptic mitochondria, which supply the ATP needed as a source of energy for these functions (Petersen et al., 2019).

Huntington’s disease is a rare progressive hereditary neurodegenerative disorder whose symptoms include uncontrolled movements, cognitive decline, and psychiatric issues. It is caused by a mutation in the huntingtin gene (HTT), which causes an expansion of a glutamine-containing repeat sequence (polyQ) beyond 36 repeats. Htt inclusion bodies are composed of amyloid fibrils. One way in which Htt aggregates could cause toxicity is through depleting ubiquitin, a protein that binds to misfolded proteins to promote their clearance through the ubiquitin-proteasome system (Ciechanover and Schwartz, 1998). Folger and Wang propose a model that “misfolded protein aggregation soaks up free ubiquitin like a sponge, leading to cytotoxicity by slowing ubiquitin-dependent proteasomal degradation of other proteins” (Folger and Wang, 2021).

Notably, the N-terminal 17-residue domain stretch of huntingtin folds into an amphipathic α-helix in the presence of membranes (Pandey et al., 2018). N-terminal fragments form aggregates and induce neuritic degeneration in cultured striatal neurons (Li et al., 2000). There appear to be three distinct aggregation pathways for Huntingtin: (1) unseeded in solution, (2) seeded in solution, and (3) membrane-mediated. Initiation of aggregation into an oligomer requires an expanded polyQ region and an *α*-helix formation at the N-terminus. *β*-sheets form in the second phase. The slowest step is the structural maturation of the proline-rich domain. Seeding accelerates aggregation and is a prerequisite for prion-like spreading. Remarkably, membranes can catalyze aggregation even more potently than seeding, and this also causes extensive membrane damage. The N-terminal *α*-helix governs membrane-mediated aggregation by anchoring the protein into the membrane (Pandey et al., 2018). Similar phenomena of membranes promoting *α*-helix formation followed by membrane damage have been observed for at least two other amyloidogenic proteins: *α*-synuclein, linked to Parkinson’s disease (Dikiy and D Eliezer, 2012) and IAPP, associated with diabetes (Jayasinghe and Langen, 2005; Jayasinghe and Langen, 2007).

Mitochondrial dysfunction in the striatum is a primary feature of Huntington’s disease (Oliveira, 2010). In Huntington’s disease, mutant Htt accumulates in the synapses in the striatum, where it is often associated with mitochondria (Petersen et al., 2019). Mitochondria in striatal synapses in mice with mutated Htt show an increased rate of proton leakage compared to wild type mice. This is associated with a reduced capacity to handle large calcium loads and increased depolarization in response to calcium (Petersen et al., 2019). Researchers have also noted significantly decreased ATP levels in synaptosomes isolated from the forebrains in a mouse model of Huntington’s disease (Orr et al., 2008). It is tempting to speculate that mutant Htt entering mitochondrial membranes via the N-terminal *α*-helix in the synapses within the striatum is an important factor facilitating mitochondrial dysfunction.

While Huntington’s disease is widely considered to be due to a gain-of-function toxic effect, it has been demonstrated through selective deletion of Htt in striatal projection neurons of mice that these neurons require Htt for motor regulation, synaptic development, cell health, and survival during aging (Burrus et al., 2020). This suggests that a loss-of-function mechanism cannot be ruled out.

Free copper enhances the aggregational toxicity of Htt (Lobato et al., 2024). Elevation of copper and iron in the brain striata has been found at early stages in Huntington’s disease (Rosas et al., 2012; Pfalzer et al., 2022). Copper plays an important role in the depletion and oligomerization/aggregation of wild type Htt. The addition of equimolar or higher concentrations of Cu^2+^ to Htt induces oligomerization, along with the rapid reduction of Cu^2+^ to Cu^1+^ (Neupane et al., 2025). Histidine residues 82 and 98 are both essential for copper interaction, and they appear to coordinate a single copper ion. Thus, Htt reduces Cu^2+^ to Cu^1+^, and copper promotes its aggregation, mediated by histidine residues (Fox et al., 2007).

**FIGURE 6:**
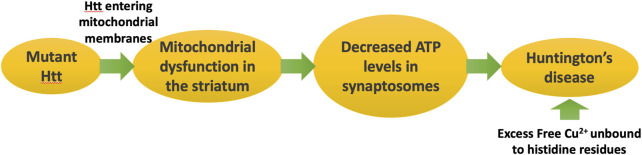
Huntington’s disease is caused by a genetic defect in Huntingtin (Htt), which leads to formation of mitochondrial membrane pores, disrupting ATP synthesis. Deficiencies in ATP in the synaptosome lead to symptoms of Huntington’s disease. Huntingtin traps copper and reduces it from Cu^2+^ to Cu^1+^, potentially protecting from copper toxicity.

Figure 6 schematizes the pathways that lead to symptoms of Huntington’s disease as a consequence of misfolded mutant Htt.

### 10.1 Multiple proteins interact with cardiolipin to induce pore formation

While several amyloidogenic proteins have been known to disrupt the plasma membrane through pore formation, recently there has been growing interest in the concept that pore formation in the mitochondrial membrane may be the primary factor that initiates neurodegenerative disease, as well as type II diabetes. An emerging concept is that cardiolipin migration to the outer membrane of the mitochondrion is the trigger that induces misfolding into soluble oligomers of various proteins present in the cytoplasm. Subsequent pore formation following binding to cardiolipin may then facilitate entry of the oligomers into the intermembrane space, where they then gain access to much more cardiolipin present on the inner membrane. This can launch an apoptotic cascade through the release of cytochrome c, resulting in neuronal cell death. A recent review paper provides a great deal of detail about various pathways where cardiolipin plays a central role in driving pore formation in a variety of different pathogenic mechanisms (Vassallo, 2025).

**FIGURE 7:**
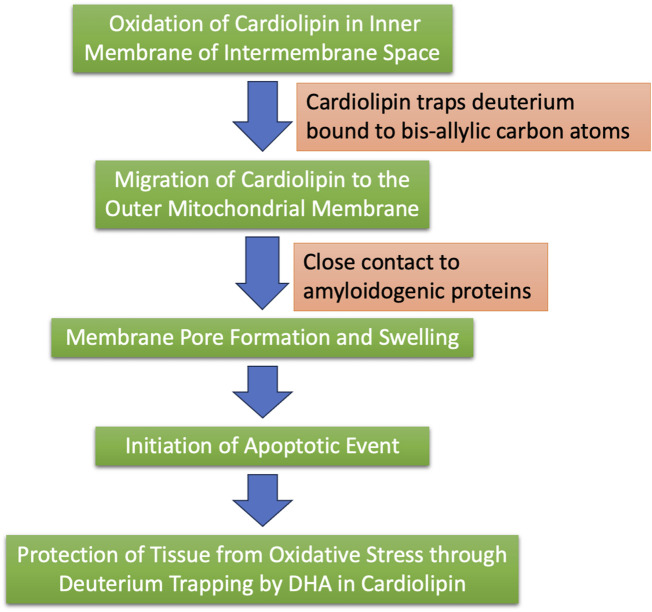
Schematic of the process by which we hypothesize that cardiolipin, synthesized in the mitochondria, may trap deuterium and ultimately sequester it in plaque containing amyloid proteins.

Figure 7 illustrates schematically the steps that we propose take place over a long period of time, with cardiolipin initially protecting the ATPase pumps from deuterium, but ultimately, as the diseased state progresses, sequestering deuterium trapped in cardiolipin along with amyloid deposits.

Bax is a Bcl-2 family protein that plays a crucial role in apoptosis. Research has shown that Bax by itself is capable of inducing pore formation in the mitochondrial membrane, following interaction with cardiolipin (Lai et al., 2019). Cytochrome c also binds to cardiolipin, and the Cytochrome c-cardiolipin conjugate opens up lipid pores in the membrane, resulting in the release of cytochrome c into the cytoplasm, thus facilitating its own escape and initiating an apoptosis cascade (Bergstrom et al., 2013).

Table 1 provides a list of proteins that can form oligomers and interact with cardiolipin to induce pore formation and disease processes, with associated references.

**TABLE 1:** Examples of proteins that have been shown to be induced to form pores in the mitochondrial outer membrane when exposed to cardiolipin.

Protein	Consequence	References
Prion protein	Creutzfeldt Jakob disease	Yang et al. (2023)
Alpha-synuclein	Parkinson’s disease	Ghio et al. (2019)
Tau	Alzheimer’s disease	Camilleri et al. (2020)
Huntingtin	Huntington’s disease	Adegbuyiro et al. (2021)
IAPP	Type II diabetes	Gurlo et al. (2010)
BAX	Apoptosis	Lai et al. (2019)
Cytochrome c	Apoptosis	Bergstrom et al. (2013)
Gasdermin D	Endotoxemia	Tang et al. (2024)

## 11 Conclusion

In this paper, we have investigated the role of deuterium in mitochondrial dysfunction, and the potential role of amyloidogenic proteins in sequestering deuterium within plaque deposits, both in the brain and in the pancreas. We have also considered the important role of copper, both as a catalyst and as an inducer of oxidative stress. We propose that amyloidogenic proteins may protect from copper overload and facilitate copper delivery to the enzymes that depend on it. Copper may also be sequestered, along with deuterium, in the plaque deposits, under conditions of copper overload, which is toxic for the cell.

Gut microflora, by supplying deuterium depleted nutrients to the host, safeguard normal mitochondrial function in the organism. Furthermore, bis-allylic carbon-containing PUFAs, such as the ones found in cardiolipin, may function as a filter in the inner membrane of the mitochondrial intermembrane space to trap deuterium before it reaches the ATPase pumps, protecting the cells from mitochondrial-respiratory chain dysfunction.

**FIGURE 8:**
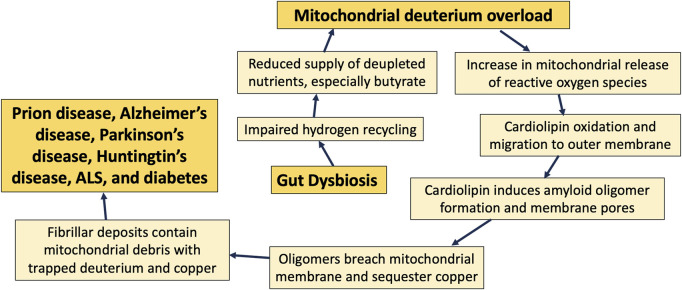
Schematic of the sequence of events that transpire over decades, beginning with an imbalance in gut microbes and eventually leading to neurodegenerative disease.

Figure 8 summarizes the main points of this paper, where we claim that excessive levels of deuterium in the mitochondria due to gut dysbiosis may be the driving force that ultimately results in cardiolipin oxidation and migration to the outer membrane, following its accumulation of deuterium bound to the bis-allylic carbon atoms. Amyloidogenic proteins then interact with cardiolipin in the outer membrane to induce an apoptotic cascade, resulting in neuronal cell death and, ultimately, neurodegenerative disease.

Cardiolipin migrates to the outer membrane when it becomes oxidized, and it may ameliorate oxidative stress there through prior deuterium doping. Cardiolipin in the outer membrane also facilitates the transformation of multiple amyloidogenic proteins into pore-forming oligomers that can disrupt the membrane potential, leading to destruction of the mitochondrion, and, in the extreme case, initiation of an apoptotic cascade. Mitochondrial membrane debris is commonly found in plaque deposits in the brain, and we hypothesize that the fibrils sequester deuterium-doped lipids, resulting in reduced deuterium burden in the cell and the tissue.

Copper acts as a catalyst in critical proteins involved in oxidative phosphorylation and antioxidant defenses in the mitochondria. The process of safely delivering copper to these enzymes is complex, and disruption of this process can lead to disease, particularly neu-rodegenerative disease.

Histidine residues in amyloid proteins play an essential role in copper homeostasis. The copper interaction anomalies in these proteins contribute to misfolding and aggregation that is linked to neurodegeneration. Impaired copper and deuterium homeostasis has neurotoxic effects via amyloidogenic proteins in prion disease, Parkinson’s disease, Alzheimer’s disease, ALS, and Huntington’s disease. Similar mechanisms of amyloidogenesis in the pancreas contribute to the development of diabetes.

Pure speculation based on experiments with heavy water suggests that histidine may be able to trap deuterium in its imidazole ring, and copper may serve as a catalyst for this by inducing proton currents. However, we were unable to find any literature on the notion that histidine traps deuterium under physiological conditions. This remains a topic for future investigation.
