# Supplementary figures and images for: Deuterium trafficking, mitochondrial dysfunction, copper homeostasis, and neurodegenerative disease
Source: Front Mol Biosci. 2025 Jul 22;12:1639327. doi: 10.3389/fmolb.2025.1639327 (PMC12322706; doi:10.3389/fmolb.2025.1639327)

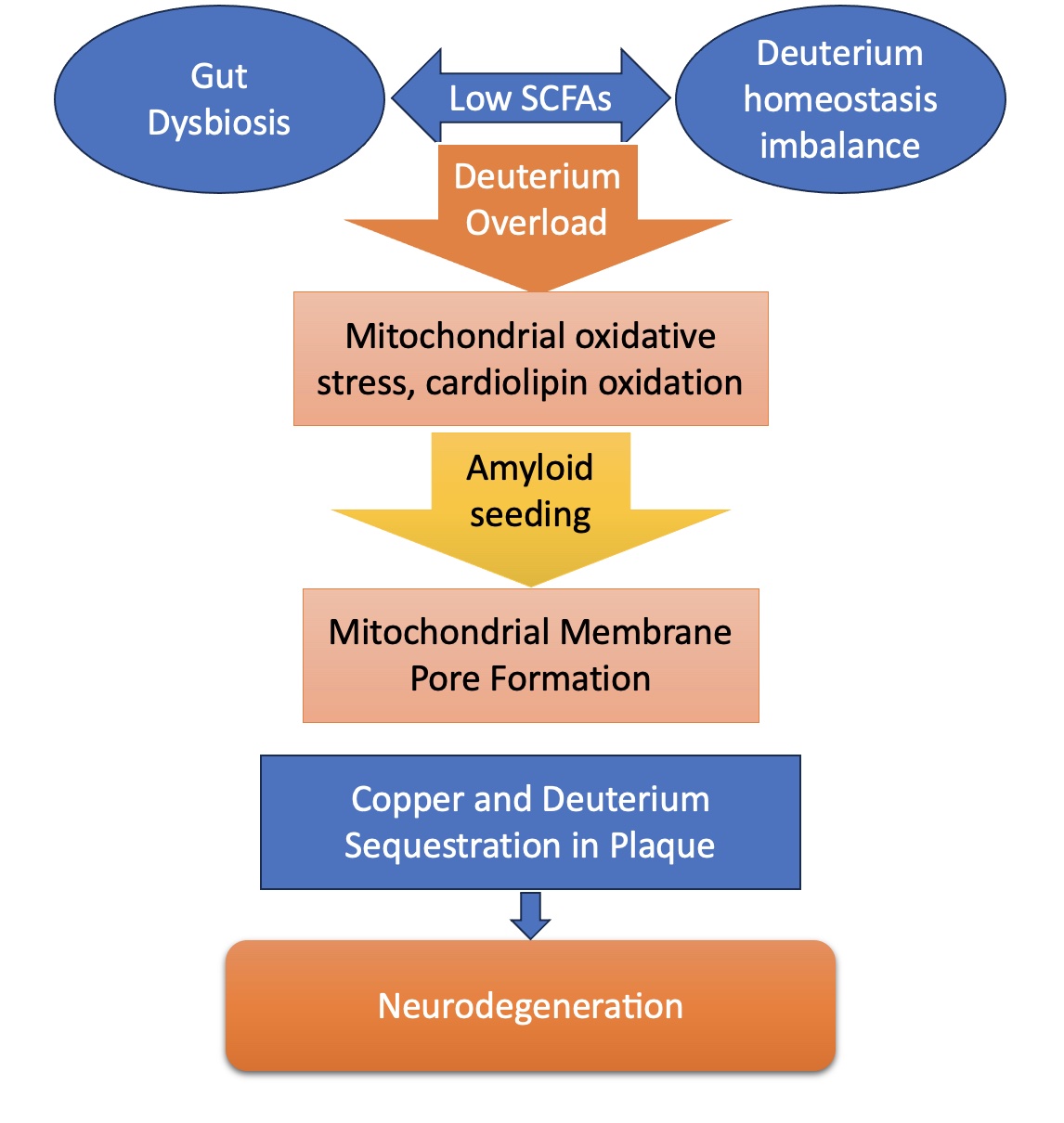

Supplement: Supplementary file 1 [file Image1.jpeg]
